# Electric Field-Assisted Delivery of Photofrin to Human Breast Carcinoma Cells

**DOI:** 10.1007/s00232-013-9533-z

**Published:** 2013-04-02

**Authors:** Joanna Wezgowiec, Maria B. Derylo, Justin Teissie, Julie Orio, Marie-Pierre Rols, Julita Kulbacka, Jolanta Saczko, Malgorzata Kotulska

**Affiliations:** 1 Institute of Biomedical Engineering and Instrumentation, Wrocław University of Technology, Wybrzeze Wyspianskiego 27, 50-370 Wrocław, Poland; 2 CNRS, Institut de Pharmacologie et de Biologie Structurale (IPBS), 205 route de Narbonne, 31077 Toulouse, France; 3 Université de Toulouse, UPS, IPBS, 31077 Toulouse, France; 4 Department of Medical Biochemistry, Wrocław Medical University, Chalubinskiego 10, 50-368 Wrocław, Poland

**Keywords:** Breast cancer cells, Electropermeabilization, Electroporation, Photodynamic reaction, Photofrin

## Abstract

The influence of electroporation on the Photofrin uptake and distribution was evaluated in the breast adenocarcinoma cells (MCF-7) and normal Chinese hamster ovary cells (CHO) lacking voltage-dependent channels in vitro. Photofrin was used at a concentration of 5 and 25 μM. The uptake of Photofrin was assessed using flow cytometry and fluorescence microscopy methods. Cells viability was evaluated with crystal violet assay. Our results indicated that electropermeabilization of cells, in the presence of Photofrin, increased the uptake of the photosensitizer. Even at the lowest electric field intensity (700 V/cm) Photofrin transport was enhanced. Flow cytometry results for MCF-7 cells revealed ~1.7 times stronger fluorescence emission intensity for cells exposed to Photofrin and electric field of 700 V/cm than cells treated with Photofrin alone. Photofrin was effective only when irradiated with blue light. Our studies on combination of photodynamic reaction with electroporation suggested improved effectiveness of the treatment and showed intracellular distribution of Photofrin. This approach may be attractive for cancer treatment as enhanced cellular uptake of Photofrin in MCF-7 cells can help to reduce effective dose of the photosensitizer and exposure time in this type of cancer, diminishing side effects of the therapy.

## Introduction

Photodynamic therapy (PDT) is a treatment modality applied in a number of cancer and noncancer diseases. It involves administration of a photosensitive agent (called photosensitizer), which is activated with a light of a specific wavelength. Reactive oxygen species (ROS) are generated and interact with cellular components, leading to oxidative stress and cell death. Therapeutic effect of PDT results from direct cytotoxicity, vascular damage and immunological response. The mode of cell death depends on several factors, such as properties of a photosensitizer, cell type, drug and light dose. Selectivity of PDT is achieved by the localized delivery of light and localized accumulation of a photosensitizer (Agostinis et al. 2011; Choudhary et al. 2009; Stamati et al. 2010; Robertson et al. 2009).

The ideal photosensitizer accumulates selectively in a tumor tissue, and has a high photocytotoxicity but minimal dark toxicity. It should also be efficiently removed from the body. Photosensitizer uptake and localization is particularly important for the resulting photodamage, due to short migration distance of singlet oxygen. To improve transport of the photosensitizer, several systems have been proposed: quantum dots (Samia et al. 2006), emulsions (Marchal et al. 2007), liposomes, nanoparticles (Josefsen and Boyle 2008) and methods such as ultrasounds, iontophoresis, electrophoresis and electroporation (for a review, see Donnelly et al. 2009; Juzeniene et al. 2007; Kotulska et al. 2013).

Electroporation (EP, electropermeabilization) is a reversible process of transient increase of the cell membrane permeability, due to exposure to external electric field pulses of high intensity. This technique, depending on the applied pulses parameters, may be used in many different disciplines. The most-developed area of its applications is medicine. Reversible EP can be used for enhancement of anticancer therapies. A combination of EP with chemotherapy is called electrochemotherapy (ECT) and it is already successfully used in clinical practice to overcome the problem with drug delivery. Two chemotherapeutic drugs are the best candidates for ECT: bleomycin and cisplatin (Gehl 2003; Kotulska 2007; Miklavcic et al. 2012; Mir 2006; Rols 2006; Serša et al. 2008). Another method, electrogene therapy, is currently under preclinical trials. It is an electrically assisted nonviral method of nucleic acid delivery (Chabot et al. 2013; Rols 2010; Sukharev et al. 1992). Irreversible EP has been proposed as a method of nonthermal, minimally invasive ablation (Davalos et al. 2005). In addition to numerous medical applications, EP is a very useful technique for biology, biotechnology and food industry (Kanduser and Miklavcic 2008).

In the area of PDT the vast majority of research studies new photosensitizers, and very few are focused on innovative systems for photosensitizers delivery. Several studies considered combination of EP with PDT for enhancement of the photosensitizers transport. Labanauskiene et al. (2009) demonstrated that EP improved an access of two photosensitizers: chlorine e6 (C e6) and aluminium phthalocyanine tetrasulfonate (AlPcS4) to murine hepatoma MH22A cells. EP-enhanced cellular uptake of photosensitizers has a significant impact on the viability of cells. Ward et al. (1997) described the effects of electric-field enhanced activation of hematoporphyrin derivate (HpD) on HeLa cells. The results demonstrated an increased degree of cell lysis, even in nonirradiated cells. The authors suggest that some form of HpD activation event was occurring during the application of the electric pulses. Lambreva et al. (2004) demonstrated that EP of cell membrane supported penetration of macromolecular chromophore dextrans acting as photosensitizers. The human histolytic lymphoma U-935 cells and the human chronic myeloid, leukemia K-562 cells reach high ratios of necrotic cells. To overcome one of the major drawbacks of systemic administration and to reduce drug dose, Johnson et al. studied potential improvement of local delivery of photosensitizers. As a result, with pulsed electric field delivery, almost all amount of the drug was delivered to the target region, reducing the systemic toxicity and time of incubation. The authors believe that enhanced cutaneous delivery of methylene blue and ALA was due to the combination of de novo permeabilization of the stratum corneum, passive diffusion through the permeabilization sites, and electrophoretic and electro-osmotic transport (Johnson et al. 1998, 2002). In our previous studies, we assessed the influence of EP on PDT with HpD, which is a less purified product than Photofrin (Kulbacka et al. 2011).

Photofrin (porfimer sodium) is a commercial, purified hematoporphyrin derivative. This is a mixture of compounds including hematoporphyrin monomers, dimmers and oligomers and it has not been fully characterized. Photofrin is a first generation photosensitizer approved for clinical applications and used to treat a variety of tumors, with successful therapeutic results. However, the main disadvantage associated with Photofrin is prolonged skin photosensitivity and relatively low specificity for tumor tissue (Choudhary et al. 2009; Samia et al. 2006; Berg et al. 2011; Ferreira et al. 2009).

The concept of Photofrin-mediated PDT enhanced with EP has not been studied yet; however, there are many studies on Photofrin-mediated PDT (Hajri et al. 2002; Luo et al. 2010; Tong et al. 2000; Chang et al. 2008; Korbelik and Krosl 1996; Henderson et al. 2000; Schweitzer 2001; Jiang et al. 1998; Kulbacka et al. 2010). Several studies on Photofrin transport and accumulation mechanism were also conducted. Due to its hydrophobicity, Photofrin concentrates in the mitochondria, endoplasmic reticulum, cytoplasmic and nuclear membrane and perinuclear region of the cytoplasm of cells in vitro. A potentially important target for PDT is cardiolipin—a phospholipid found in the inner membrane of mitochondria and at the contact sites between the inner and outer membranes. It was demonstrated that lipophilic sensitizers are taken up by cells following an LDL receptor-mediated endocytosis (Teiten et al. 2001; Peng et al. 1996; Rodriguez et al. 2008; Morgan and Oseroff 2001; Chwilkowska et al. 2003; Wilson et al. 1997). After a brief incubation (3 h), the main target site of Photofrin is plasma membrane. After a prolonged incubation (24 h), it moves to intracellular compartments: the Golgi complex, mitochondria, lysosomes—the specific pattern of localization depends on the cell type (Chang et al. 2008; Wilson et al. 1997; Hsieh et al. 2003).

Enhancement of Photofrin delivery by EP may not only change the amount of incorporated photosensitizer, but also the time and the site of its accumulation. It is particularly significant as the type of cell death depends on the localization of a photosensitizer in cells. When plasma membrane is the main target, upon irradiation the cell death phenotype is necrosis like (Chang et al. 2008; Hsieh et al. 2003). Dellinger showed that short-time incubation of cells with high concentrations of Photofrin results in a leakage of cytoplasm through photodamaged membranes, while longer incubation with low concentration of Photofrin leads to apoptotic response (Dellinger 1996). In our previous work we described distribution of Photofrin in several cell lines, including MCF-7 cells. We observed the most intensive signal around the nuclear envelope after 4 h of incubation. Photofrin-mediated PDT caused immediate cell death via apoptosis (Saczko et al. 2007, 2008).

Considering in vivo conditions, it was reported that both Photofrin-induced PDT alone and ECT alone were effective in breast cancer treatment. Photofrin-induced PDT, applied to breast cancer patients with chest wall progression, gave high response ratios allowing good long-term local tumor control (Cuenca et al. 2004). In other studies a high efficiency and a good safety profile of ECT with cisplatin or bleomycin, as an alternative approach for a treatment of chest wall breast cancer recurrence or cutaneous tumor lesions of breast cancer, were demonstrated (Rebersek et al. 2004; Sersa et al. 2012). Encouraged with these reports, we decided to investigate if a combination of Photofrin-induced PDT with EP may be an effective approach for breast cancer treatment. Additionally, for a comparison, we applied hamster ovarian cells lacking voltage-dependent channels, which are often used as reference cells in ECT. If electric pulses can enhance cellular uptake of Photofrin, combining PDT with EP would help to reduce effective dose of the photosensitizer and time of its accumulation, diminishing side effects of the therapy.

## Methods

### Chemicals

Photofrin was purchased from QLT PhotoTherapeutics, Inc., Vancouver, Canada. 10 mM stock solution was prepared in MCF-7 culture medium. The final concentrations were obtained by direct dilution of the stock solution in the culture medium or EP buffer with low electrical conductivity (10 mM phosphate, 1 mM MgCl_2_, 250 mM sucrose, pH 7.4).

### Cell Culture

The studies were performed on human breast adenocarcinoma cell line (MCF-7). Additionally, Chinese hamster ovary cells (CHO-WTT) were a model for transport studies on EP due to very low expression of endogenous ion channels (Gamper et al. 2005). MCF-7 cells were grown in RPMI 1640 (Eurobio, France) supplemented with 10 % fetal bovine serum (Lonza, Switzerland), l-glutamine (Life Technologies, USA) and penicillin/streptomycin (Eurobio, France). MCF-7 cells were grown as a monolayer plated on a plastic flask 75 cm^2^ (Nunc, Denmark) and detached for the experiments by trypsinization (trypsin 0.025 % EDTA 0.02 % solution, Eurobio, France). CHO cells were grown in MEM (Eurobio, France) with appropriate supplementation, according to (Rols et al. 1995). CHO cells were grown in suspension in a spinner flask (100 ml), placed on a stirring unit (IKA, Germany). Cells concentration was maintained in the range of 0.5–0.6 ×10^6^ cells/ml. Cell culture was performed in a humidified atmosphere at 37 °C and 5 % CO_2_.

### Protocols

#### Spectra of Photofrin

Solution of Photofrin in PBS with a concentration of 5 μM (Eurobio) was prepared. Spectra of fluorescence excitation and emission were recorded with QM-4, Photon Technology International. Fluorescence excitation spectrum was recorded for the wavelength of emission of 615 nm. Fluorescence emission spectrum was recorded for the wavelength of excitation of 500 nm.

#### Electroporation Procedure

After trypsinization and centrifugation (5 min, 800 rpm, Centrifuge 5702R Eppendorf), cells were counted and for each sample of 0.5 × 10^6^ cells they were resuspended in 100 μl of EP buffer (10 mM phosphate (Sigma), 1 mM MgCl_2_ (Sigma), 250 mM sucrose (Sigma), pH 7.4). Cell suspension was pulsed in 35 mm Petri dishes (Nunc, Denmark), between two stainless-steel parallel plate electrodes, 4 mm distant. EP was performed using Betatech S20 (Betatech, L’union, France), which delivered five rectangular electrical pulses (different values of amplitude, pulse duration of 50 μs, frequency of 1 Hz). The oscilloscope Enertec 5026 monitored the pulses. After pulsation, cells were left for 2 min at room temperature.

#### Viability Assay

Viability of electropermeabilized cells was assessed in the following way: 2 ml of culture medium was added and cells were grown in Petri dishes for 24 h at 37 °C in 5 % CO_2_. Cell viability was determined by coloration, using crystal violet (Sigma) method. Cells were rinsed with PBS, incubated for 20 min with crystal violet solution, rinsed three times with PBS and incubated for 10 min with 10 % acetic acid. After shaking, 50 μl of cells were dissolved in acetic acid with 1 ml of Milli-Q water. The absorbance of each sample was measured at 595 nm using Novaspec II, Pharmacia Biotech. The results were expressed as the percentage of viability, relative to untreated control cells (cells to which no dye was added and no pulses were applied). Three samples were prepared per each experiment. Mean values and standard deviations were calculated.

#### Electropermeabilization Efficiency: Propidium Iodide Uptake

Electropermeabilization efficiency was assessed by the penetration of impermeant dye—propidium iodide. Immediately before EP, the cells were exposed to 100 μM propidium iodide (PI, P4170, Sigma). Two minutes after pulsation cells were resuspended in 1 ml of PBS. Samples were analyzed with a FACS Calibur flow cytometer (Becton–Dickinson) immediately after permeabilization.

#### Electropermeabilization Efficiency: Photofrin Uptake

Electropermeabilization of cells was quantified by the penetration of Photofrin. Immediately before EP, the cells were exposed to 25 μM Photofrin. Two minutes after pulsation cells were resuspended in 1 ml of PBS. Samples were analyzed immediately after permeabilization. Cellular uptake of Photofrin was examined under a fluorescent microscope and a flow cytometer.

##### Fluorescent Microscopy Studies

Cells were observed under MacroFluo Leica Z16 APO with PLANAPO 5.0×/0.50 LWD objective and 9.2 magnification (Leica, Germany). Exposure time was 10 ms (white light) and 1000 ms (fluorescent light). Cells were excited at 587 nm, fluorescence emission was read at 610 nm. Three images per condition were recorded. Images were analyzed with ImageJ software. The same brightness scale was set for all images in one experiment. Three-dimensional profiles of each single cell were obtained by the use of Interactive 3D Surface Plot Plugin to ImageJ software. Mean gray scale values were computed for each single cell in each fluorescent image separately and they were considered as equivalent of the fluorescent emission intensity of each single cell. Statistical analysis was performed with MATLAB 2011b (MathWorks). Statistical distribution of mean gray values was described with box plots. One box was created for each condition (each value of electric field intensity). In each box, the central mark is median, the edges of the box are the 25 and 75th percentiles, the whiskers extend to the most extreme data points not considered outliers, and outliers are plotted individually (marked with crosses).

##### Flow Cytometry Analysis

Each sample was transferred to tube (Starlab, Switzerland) in ice and analyzed by flow cytometry (FACSCalibur, Becton–Dickinson) to determine the efficiency of electropermeabilization (the level of fluorescence associated with electropermeabilization). The samples were excited using the 488 nm line of an argon laser and detection of fluorescence was performed in FL-2 channel (for PI detection) or FL-3 channel (for Photofrin detection). Light-scatter and fluorescence measurements were used as an indication of an object size and shape, allowing for discrimination between cells, microspheres and debris. Data were analyzed using CellQuest software (Becton–Dickinson) and presented as the geometric mean of fluorescent emission intensities of the positive cells.

#### Photodynamic Reaction Supported with Electroporation (EP–PDR)

Before electric pulses delivery, proper volumes of Photofrin solution were added to the EP buffer. Then EP was performed. The whole experiment was conducted in dark conditions. One hour after EP cells were irradiated. Red (the whole spectrum from the range of ~615–675 nm with the peak at 640 nm), green (the whole spectrum from the range of ~475–600 nm with the peak at 525 nm) and blue (the whole spectrum from the range of ~430–510 nm with the peak at 458 nm) LED bulbs (2 W of power) were used as a light source. When cells were irradiated with mixed light, three lamps simultaneously delivered red, green and blue light, so the power of light was three fold greater. Samples were irradiated for 10 min in sterile conditions. Petri dish without a cover was placed on a mirror to double the light. Viability of cells was measured 24 h after the end of experiment. Dark EP–PDR experiment was a non-irradiated control for EP–PDR.

### Statistical Analysis

The results of the crystal violet assay and FACS analysis were reported as mean ± standard deviation. The significance of the difference between mean values of different groups of cells was assessed by Student’s *t* test with *p* value of *p* ≤ 0.05 or *p* ≤ 0.005, to show the statistical significance.

## Results

### Spectra of Photofrin

**Fig. 1:**
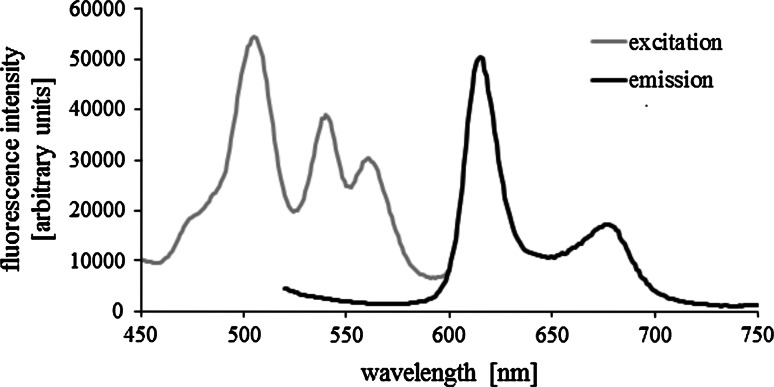
Fluorescence excitation and emission spectra of Photofrin (5 μM in PBS)

Fluorescence spectra of Photofrin are presented in Fig. 1. The maximum of excitation occurred at approximately 500 nm, two other peaks were measured at 540 and 560 nm. The maximum of emission occurred at 615 nm, the second peak was measured at ~680 nm. Recorded spectra were used to select a proper wavelength range for Photofrin excitation.

### Sensitivity to Electropermeabilization

**Fig. 2:**
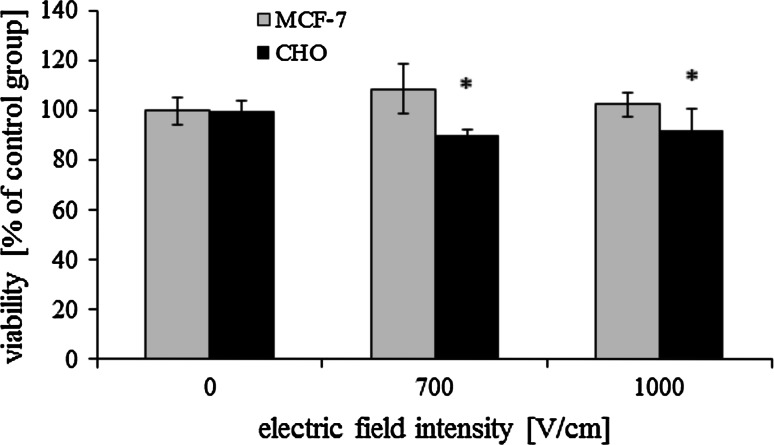
Viability of CHO and MCF-7 cells after electroporation (5 pulses with a duration of 50 μs were delivered at the frequency of 1 Hz; crystal violet method was performed 24 h after electroporation); *p < 0.05

Sensitivity to electropermeabilization was evaluated for both cell lines and presented in Fig. 2. The electric field in the studied range of intensities was not toxic for MCF-7 cells. The viability even after EP at 1000 V/cm was unaffected. CHO cells viability slightly decreased with electric field intensity. When cells were electroporated at 1000 V/cm, their viability was still high-ca. 90 %.

### Electropermeabilization Efficiency: Propidium Iodide Uptake

**Fig. 3:**
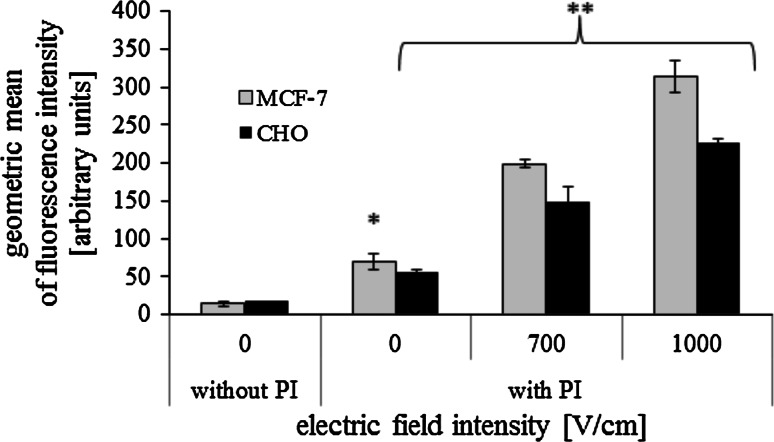
Geometric mean of fluorescence intensity measured in cells electropermeabilized with 100 μM propidium iodide (flow cytometry results; 5 pulses with a duration of 50 μs were delivered at a frequency of 1 Hz); *p < 0.05, **p < 0.005

Efficiency of MCF-7 and CHO cells electropermeabilization was assessed by propidium iodide uptake. Figure 3 presents flow cytometry results. Both cell lines revealed enhanced PI accumulation after electric pulses delivery. Geometric mean of fluorescence intensity increased with electric field intensity. Particularly high fluorescence was measured in cells electroporated at 1000 V/cm. The whole population was shifted towards higher fluorescence values.

### Electropermeabilization Efficiency: Photofrin Uptake

**Fig. 4:**
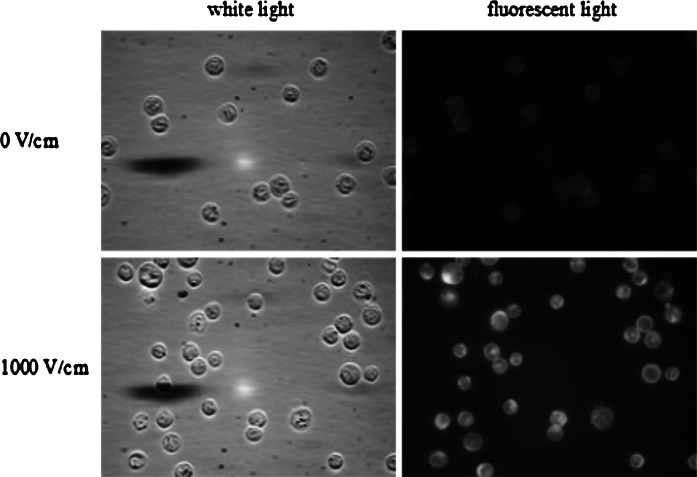
CHO cells electropermeabilized with 25 μM Photofrin (fluorescent microscopy results; 5 pulses with a duration of 50 μs were delivered at a frequency of 1 Hz)

Images of CHO cells with Photofrin are presented in Fig. 4. These results show enhanced Photofrin uptake after electric pulses application. For cells electroporated with Photofrin at 1000 V/cm, strong fluorescence intensity was observed, in contrast to non-electroporated cells.

**Fig. 5:**
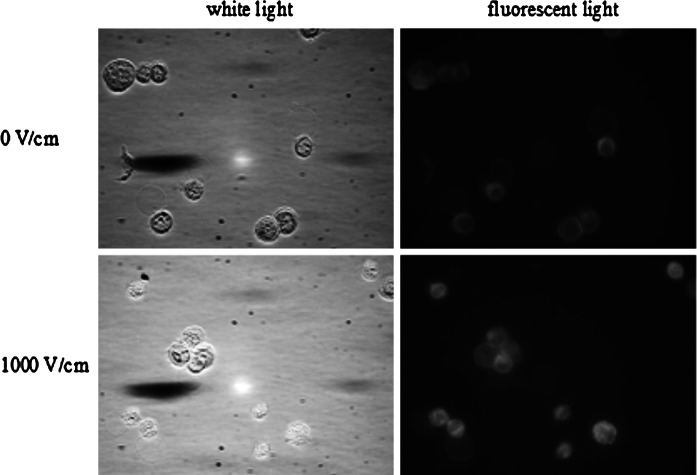
MCF-7 cells electropermeabilized with 25 μM Photofrin (fluorescent microscopy results; 5 pulses with a duration of 50 μs were delivered at a frequency of 1 Hz)

For MCF-7 cells, the fluorescence intensity of electroporated cells was also higher than the intensity of non-electroporated cells (Fig. 5). More molecules of Photofrin entered the cell after the cell membrane permeabilization.

**Fig. 6:**
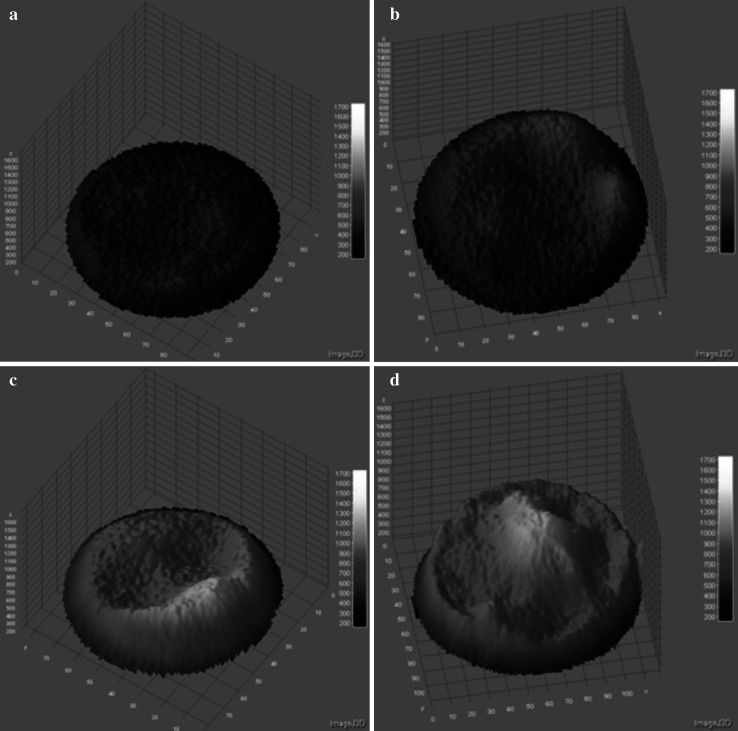
3D profiles of CHO cells electropermeabilized with 25 μM Photofrin: **a** 0 V/cm, **b**, **c** 700 V/cm, **d** 1000 V/cm (fluorescent microscopy results; 5 pulses with a duration of 50 μs were delivered at a frequency of 1 Hz)

**Fig. 7:**
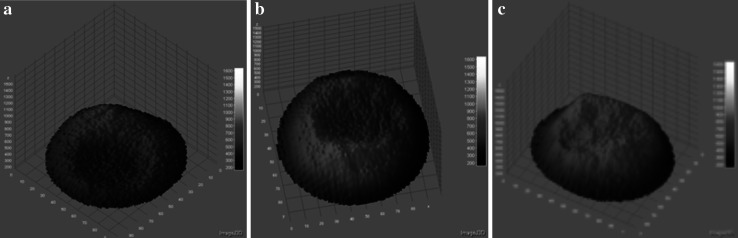
3D profiles of electropermeabilized MCF-7 cells with 25 μM Photofrin: **a** 0 V/cm, **b** 700 V/cm, **c** 1000 V/cm (fluorescent microscopy results; 5 pulses with a duration of 50 μs were delivered at a frequency of 1 Hz)

In Figs. 6 and 7, three-dimensional profiles of single cells are presented. For non-electroporated CHO cell very low fluorescence intensity was measured (Fig. 6). When cells were electroporated, fluorescence increased with the electric field intensity. For the electric field intensity of 700 V/cm, the results were not clear. One group of cells showed enhanced Photofrin accumulation (Fig. 6c), while the second one did not exhibit any significant differences in comparison with nonelectropermeabilized cells (Fig. 6b). The value of 1000 V/cm was the most effective and enabled Photofrin molecules enter the cell.

Figure 7 presents profiles of MCF-7 cells. Non-electroporated cell also did not exhibit a significant fluorescence. The fluorescence intensity detected in MCF-7 cells electroporated with Photofrin was not as high as in CHO cells; however, intensive fluorescence was observed in nuclear area of the cell.

**Fig. 8:**
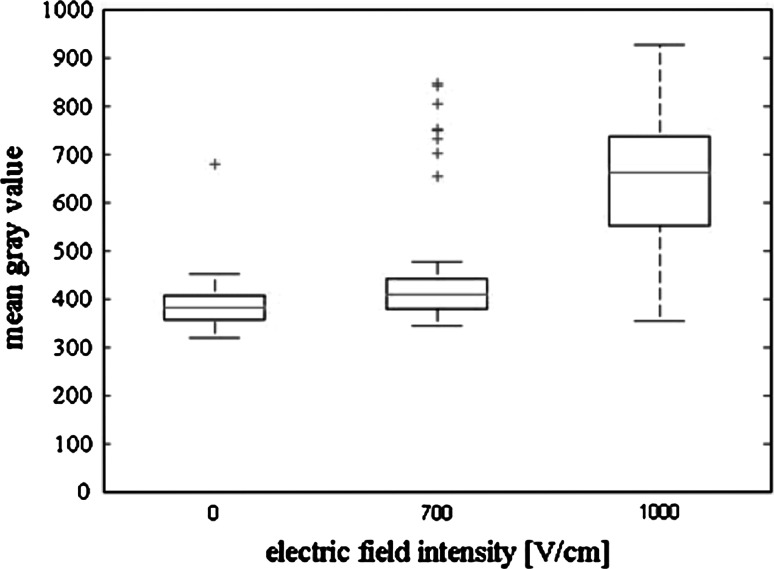
*Box plot* of mean grey value calculated for CHO cells electropermeabilized with 25 μM Photofrin (statistical parameters calculated on the basis of fluorescent microscopy results; 5 pulses with a duration of 50 μs were delivered at a frequency of 1 Hz); in each *box*, the central mark is median, the edges of the *box* are the 25 and 75th percentiles, the whiskers extend to the most extreme data points not considered outliers, and outliers are plotted individually (*crosses*)

**Fig. 9:**
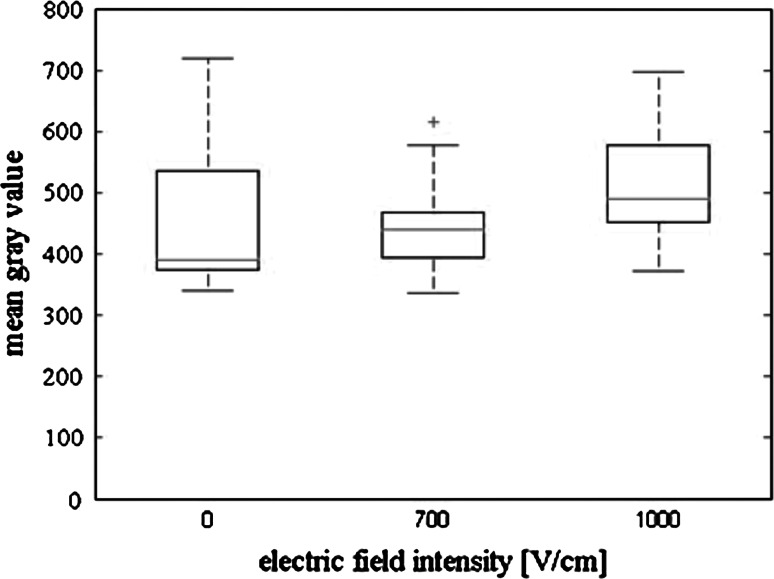
*Box plot* of mean grey value calculated for MCF-7 cells electropermeabilized with 25 μM Photofrin (statistical parameters calculated on the basis of fluorescent microscopy results; 5 pulses with a duration of 50 μs were delivered at a frequency of 1 Hz); in each *box*, the central mark is median, the edges of the *box* are the 25 and 75th percentiles, the *whiskers* extend to the most extreme data points not considered outliers, and outliers are plotted individually (*crosses*)

**Fig. 10:**
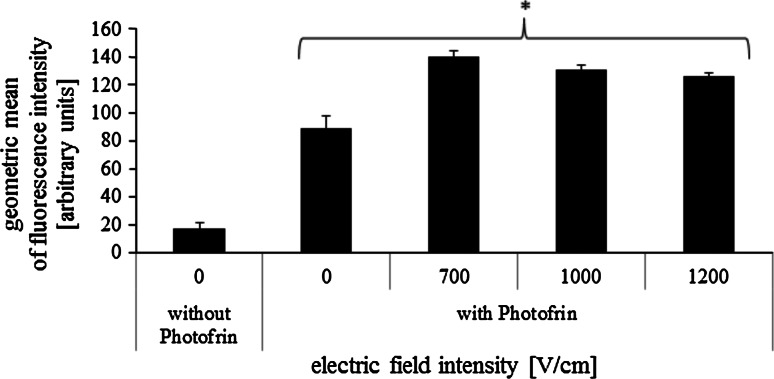
Geometric mean of fluorescence intensity of MCF-7 cells electropermeabilized with 25 μM Photofrin (flow cytometry results; 5 pulses with a duration of 50 μs were delivered at a frequency of 1 Hz); *p < 0.005

Figures 8 and 9 present statistical analysis based on the fluorescent microscopic images of cells exposed to Photofrin. For CHO cells (Fig. 8) the box plot shows an increase of median of fluorescence intensity with increasing electric field intensity. Particularly high fluorescence intensity was measured for cells electroporated at 1000 V/cm (median at the level of approximately 660 units). For non-electroporated CHO cells fluorescence intensity reached the level of approximately 380 units. For MCF-7 cells median of fluorescence intensity increased with electric field intensity (Fig. 9). The fluorescence intensity of non-electroporated MCF-7 cells was ~390 units, after EP at 1000 V/cm it increased to ~500 units. The results of flow cytometry are consistent with the previously presented microscopy images analysis (Fig. 10). Fluorescence intensity of MCF-7 cells, electroporated with Photofrin, is higher than that of non-electroporated cells exposed to the photosensitizer. However, it did not increase as significantly with electric field as it could be expected (fluorescence intensity of non-electroporated cells treated with Photofrin was at the level of 90 units, after EP at 700 V/cm it reached the level of 140 units).

### Photodynamic Reaction Supported with Electroporation (EP–PDR)

**Fig. 11:**
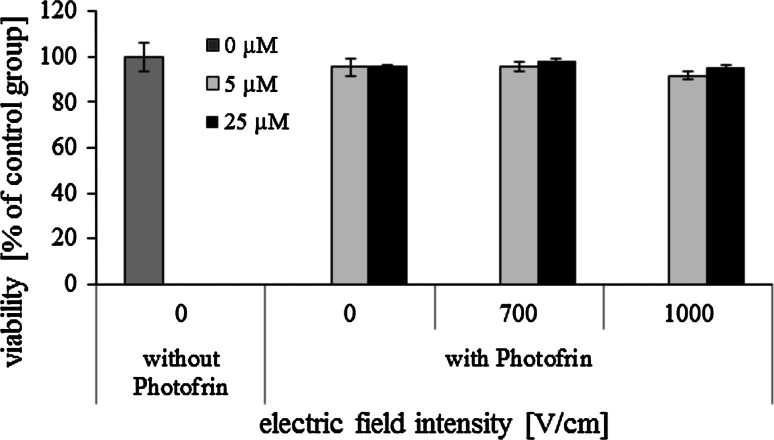
Viability of CHO cells after dark EP–PDR with Photofrin (5 and 25 μM) (5 pulses with a duration of 50 μs were delivered at a frequency of 1 Hz; crystal violet method was performed 24 h after electroporation)

The influence of EP with Photofrin on cells viability was evaluated. Figure 11 presents results of “dark EP–PDR” on CHO cells. The concentration of Photofrin and the time of incubation before irradiation used in our study (5 and 25 μM, 1 h) were lower than in standard PDT in vitro without EP. Without irradiation both studied concentrations of Photofrin were non-toxic for CHO cells. Even when pulses at electric field intensity of 1000 V/cm were applied, cells viability remained above 90 %.

**Fig. 12:**
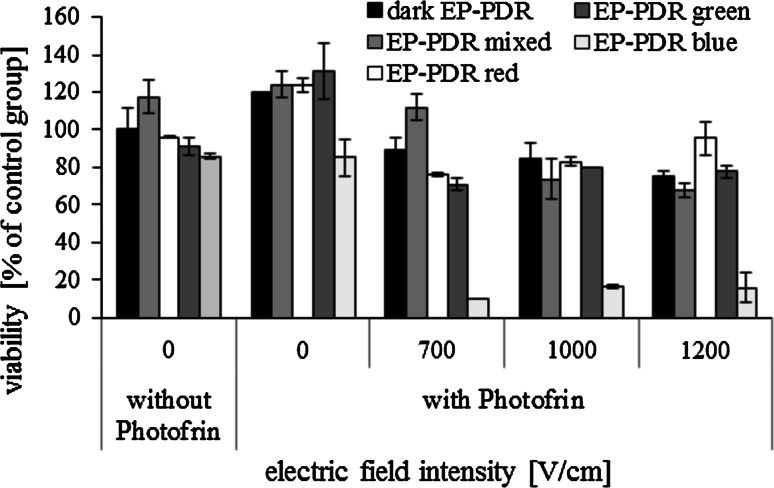
Viability of MCF-7 cells after EP–PDR with 25 μM Photofrin (5 pulses with a duration of 50 μs were delivered at a frequency of 1 Hz; cells were irradiated with *red*, *green*, *blue* and mixed light or nonirradiated; crystal violet method was performed 24 h after electroporation) (Color figure online)

Viability of MCF-7 cells electroporated with Photofrin (25 μM), both without and with irradiation, was determined. Electric field intensities of 700, 1000 and 1200 V/cm were selected. The results are presented in Fig. 12. Viability of non-electroporated cells incubated with Photofrin without irradiation and irradiated with mixed, red and green light was even higher than viability of untreated control cells. Only when cells were irradiated with blue light, their viability decreased, although it remained above 80 %. When electric pulses were additionally applied, cells viability decreased for both dark and irradiated conditions. Toxic influence of the combination of EP with Photofrin and blue light irradiation was particularly significant. Even at the lowest electric field intensity (700 V/cm) effectiveness of EP–PDR was very high—cells viability decreased below 20 %. It should be noted that Photofrin-mediated PDT of cancer cells is effective even without EP; however, in our experiment the incubation time was much shorter than what is usually applied.

## Discussion

In this work we presented an innovative approach to Photofrin-mediated PDT assisted by electric field. The results show that cells viability did not decrease after electric pulses delivery (Fig. 2), which demonstrates that EP is not toxic if appropriate parameters are used. The uptake of propidium iodide (impermeant dye) increased with electric field intensity (Fig. 3), demonstrating enhanced cell membrane permeabilization.

The main objective of this study was to observe the influence of electric pulses delivery on the Photofrin uptake and its localization in MCF-7 and CHO cells. On the basis of the fluorescence images analysis, we observed that EP of CHO cells significantly improved Photofrin uptake (Figs. 4, 6, 8). Due to electropermeabilization, Photofrin entered the cell and accumulated in the whole cell. For MCF-7 cells an increased accumulation was also observed, but not as effective as for CHO cells (Figs. 5, 7, 9, 10). It may have resulted from different properties of these cell lines. Chinese hamster ovary cells are a common model for transport studies on EP due to very low expression of endogenous ion channels (Gamper et al. 2005). In contrast, the expression of a number of voltage-gated potassium channels in MCF-7 cells has been demonstrated (Minghua and Zhi-Gang 2011; Van Tol et al. 2007; Wonderlin et al. 1995). Voltage-gated sodium channels were also identified in MCF-7 cells. Interestingly, the overall level of its expression was >100 fold higher in strongly metastatic MDA-MB-231 human breast cancer cells compared with weakly metastatic MCF-7 cells (Fraser et al. 2005). In general, ion channels attract attention of researchers as potential markers of oncogenic events and new targets of anticancer therapy (Le Guennec et al. 2007). It would be valuable to explore in detail the possible implications of expression of voltage-dependent ion channels on the uptake of drugs in MCF-7 cells.

In addition to the assessment of Photofrin transport enhancement, we also evaluated the influence of Photofrin mediated PDT, assisted by EP, on MCF-7 cells viability (Fig. 12).

Viability of non-electroporated cells incubated with Photofrin without irradiation and irradiated with mixed, red and green light was even higher than viability of untreated control cells. We assume that without EP Photofrin molecules did not manage to enter the cells due to very short time of incubation (1 h). Moreover, without irradiation (or upon irradiation with a light of improper wavelength) Photofrin was not activated and the process of cell destruction could not begin. Cell proliferation was not hampered. Maybe in the presence of drug some kind of self-defense mechanisms of cells were activated, resulting in even slightly higher viability than in the control group. Only upon irradiation with blue light did MCF-7 cell viability decrease, although it remained above 80 %. Cells viability decreased for both dark and irradiated conditions when electric pulses were additionally applied. The most efficient cell viability decrease was achieved when cells electroporated with Photofrin were irradiated with the blue light. The viability of cells electroporated with Photofrin at the lowest electric field intensity (700 V/cm) decreased below 20 % after irradiation with the blue light. Irradiation with mixed, green or red light was not effective, the same results were obtained for “dark EP–PDR” experiment. The viability of nonirradiated (or irradiated with mixed, green or red light) cells decreased when electric pulses were delivered, but it was still at the level ~70 %.

Many authors showed that Photofrin mediated PDT is effective even without EP but much longer times of incubation or higher concentrations of Photofrin should be applied: 4 h, 40 μM (Hajri et al. 2002); 16 h, 4 μM (Luo et al. 2010); 18 h, 2.5–13 μM (Tong et al. 2000); 18 h, 2–15 μM (Wilson et al. 1997); 24 h, 34 μM (Korbelik et al. 1991). In our studies cell were exposed to Photofrin only for 1 h before irradiation. EP facilitated transport of Photofrin and allowed to reduce the time required for intracellular accumulation. This reduction is of huge importance for potential clinical application since reduced time interval between drug delivery and tumor irradiation may limit skin sensitivity to sunlight, which normally occurs after Photofrin-mediated PDT.

## Conclusions

The efficiency of PDT depends on efficient uptake of the photosensitizer by cells. EP of cells enables creation of new ways of molecular transport. Using this phenomenon to photosensitizers delivery can improve PDT effectiveness and reduce drug dose. Moreover, selectivity of PDT can also be improved, as permeabilization occurs only near the area of pulses delivery. All these factors help to diminish side effects of chemotherapy.

Our study showed that the delivery assisted with electric pulses enhances Photofrin uptake by cells in vitro. A combination of photodynamic reaction with EP improved the PDR effectiveness by decrease of cell proliferation and increased nuclear accumulation of photosensitizer after EP. It is interesting that even at the low electric field intensity (700 V/cm) Photofrin transport was enhanced. Due to Photofrin anionic character, transport through ion channels or by electrophoresis may also be considered. Undoubtedly, PDT assisted by electric field is an attractive, innovative approach for cancer treatment. However, detailed studies on the mechanism of Photofrin uptake are necessary.
